# A Brief Overview of Dietary Zeaxanthin Occurrence and Bioaccessibility

**DOI:** 10.3390/molecules25184067

**Published:** 2020-09-06

**Authors:** Cristina Tudor, Adela Pintea

**Affiliations:** Department of Chemistry and Biochemistry, University of Agricultural Sciences and Veterinary Medicine, Mănăştur Street, 3-5, 400372 Cluj-Napoca, Romania; cristina.tudor@usamvcluj.ro

**Keywords:** zeaxanthin, bioaccessibility, INFOGEST, antioxidants, ocular health

## Abstract

As it exhibits no provitamin A activity, the dietary intake of zeaxanthin is not considered essential. However, its contribution to ocular health has long been acknowledged. Numerous publications emphasize the importance of zeaxanthin alongside lutein in ocular diseases such as cataracts and age-related macular degeneration which constitute an important health concern, especially among the elderly. Considering that the average dietary ratio of lutein to zeaxanthin favors the first, more bioaccessible food sources of zeaxanthin that can hinder the development and progression of the above-mentioned disorders are of great interest. In this paper, a brief overview of the more recent state of knowledge as regards dietary sources together with their respective zeaxanthin bioaccessibility assessed through a standardized in vitro digestion method was provided.

## 1. Introduction

Among the 1195 identified natural carotenoids [1], only lutein (β,ε-Carotene-3,3′-diol) and zeaxanthin (β,β-Carotene-3,3′-diol) have the ability to pass the blood–retina barrier and to accumulate in the macula lutea of the human eye. Here, the two dihydroxycarotenoids together with *meso*-zeaxanthin (a metabolite of lutein) exert their protection by filtrating high energy blue light and by limiting the oxidative stress, thus acting as powerful antioxidants [2]. The presence of lutein and zeaxanthin in the visual system relies entirely on the human diet, while *meso*-zeaxanthin, the conversion product of lutein, is believed to be formed in the retina and is rarely found in nature [3,4].

Being lipophilic pigments, lutein and zeaxanthin follow the same absorption pathway as dietary lipids. After their release from the food matrix, the oxygenated carotenoids need to be solubilized into lipid emulsion particles in the stomach, incorporated into mixed micelles stabilized by the biliary salts in the duodenum before being taken up by the small intestinal cells and packaged in chylomicrons for secretion into the lymphatic system [5]. Several proteins, such as Scavenger receptor class B type I (SR-BI), cluster determinant 36 (CD36) and Niemann-Pick C1-Like 1 (NPC1L1) have been proven to facilitate the selective uptake of carotenoids by intestinal cells, but at the same time passive diffusion of carotenoids across the enterocytes is also believed to occur in certain conditions [6,7]. The chylomicrons produced in the enterocytes and secreted into lymph are further processed by lipoprotein lipase in peripheral tissues, enter as chylomicron remnants into the bloodstream and are then transferred to the liver. Carotenoids are partially stored in the liver or packaged and secreted into circulation as very-low-density lipoproteins (VLDL), which in turn can be processed to low-density lipoproteins (LDL) [8,9]. Zeaxanthin can be secreted directly from the enterocytes within the small intestine-derived high-density lipoprotein (HDL) [10]. Contrarily to carotenes, which are primarily transported by LDL, more than 40% of the circulating zeaxanthin in the fasting state is found in the high-density lipoprotein (HDL) fraction [11]. Depending on their lipoprotein circulating form, carotenoids are taken up by peripheral cells through protein (SR-BI, CD36)-mediated processes or through LDL receptor-mediated endocytosis [9]. Despite the relatively low blood level of lutein (0.1–1.44 μmol/L in USA; 0.26–0.70 μmol/L in Europe) and especially of zeaxanthin (0.07–0.17 μmol/L in USA; 0.05–0.13 μmol/L in Europe), human retina accumulates high amounts of carotenoids (lutein, zeaxanthin and *meso*-zeaxanthin) in the macula, reaching as much as 1 mM [12,13,14]. It was established that the mechanism behind the preferential and selective accumulation of these xanthophylls in the macula involves the participation of all three human SR-B proteins (SR-B1, SR-B2 and CD36) [15,16]. Specifically, all three proteins were able to take up significant amounts of zeaxanthin and the uptake was increased in the presence of HDL. These results strengthened the idea of a direct relationship between the specific affinity of zeaxanthin for HDL and its accumulation in the retina [16]. Moreover, Bhosale et al. [17] identified a Pi isoform of glutathione S-transferase (GSTP1) as a high-affinity zeaxanthin-binding protein in the inner and outer plexiform layers of the human and monkey macula.

The amount of an ingested carotenoid that is absorbed through the gastrointestinal tract and is able to reach appropriate tissues in order to exert its biological effects is generally known as bioavailability [18]. Very often the high amount of carotenoids of a certain food source is incorrectly associated with a high bioavailability. A classic example which has been extensively researched is that of β-carotene from carrots (*Daucus carota* L.). Undoubtedly, carrots possess an elevated concentration of β-carotene but due to the entrapment of this highly lipophilic carotenoid as solid-crystalline aggregates in the chromoplasts [19,20], its release from the food matrix is hindered and a great amount of β-carotene does not become accessible (bioaccessible) for absorption by the enterocytes. Likewise, the notable amount of zeaxanthin in corn (*Zea mays* L.) [21] is not directly correlated with a high bioaccessibility [22]. The deposition form of carotenoids in plant and animal-based foods (solid-crystalline aggregates, lipid-dissolved forms or liquid-crystalline forms) exerts a strong influence on their liberation from the food matrix and consequently on their bioavailability [20]. Zeaxanthin, present (mainly as zeaxanthin dipalmitate) in liquid-crystalline form in the tubular chromoplasts of goji berries, showed an enhanced liberation and bioaccessibility compared to lutein, which is stored as protein-complexes in the thylakoids of chloroplasts [23]. Alongside the arduous liberation from foods, other factors such as the presence of co-ingested fat [24], fiber [25] or the processing level of the investigated food sample [26,27] can have a significant impact on carotenoid bioaccessibility. Attributable to its distinguished provitamin A activity, the bioaccessibility of β-carotene from foods has been broadly investigated and less emphasis has been given to the bioaccessibility of xanthophylls such as lutein and zeaxanthin. Furthermore, as lutein is the more predominant xanthophyll in natural food sources [28], its average dietary intake is considerably higher than that of zeaxanthin [29] and despite the fact that lutein, zeaxanthin and *meso*-zeaxanthin are equally present in the human macula [30], research on zeaxanthin is noticeably reduced as compared to lutein in terms of novel food sources, bioaccessibility and bioavailability. Despite the emerging evidence correlating the intake of carotenoids (including xanthophylls lutein and zeaxanthin) with a decreased risk of several disorders, there is no dietary recommendation for these lipophilic pigments [29].

Given the fact that a high amount of zeaxanthin in the micellar phase is associated with a potentially high absorption by the intestinal cells and transportation into plasma, data on the bioaccessibility of zeaxanthin from different food sources is a prerequisite for determining its bioavailability [31]. For this reason, the present review has been focused towards exploring the available information regarding the occurrence and bioaccessibility of zeaxanthin from foods of different origin. A small-scale direct comparison of the bioaccessibility results obtained within different research groups was possible due to the recent elaboration of the standardized in vitro digestion protocol [32].

We reviewed publications that reported dietary sources, bioaccessibility assessment and health benefits of zeaxanthin by using the online databases of Web of Science, Scopus and ScienceDirect. Several search terms such as zeaxanthin bioaccessibility, xanthophylls, macular pigment, INFOGEST, in vitro digestion, age-related macular degeneration and ocular health were used to obtain relevant English-language papers published from 1975 up to July 2020. Zeaxanthin bioaccessibility studies using both the standardized in vitro digestion protocol and other simulated digestion methods were included in the final analysis. Studies that reported the concentration of zeaxanthin together with lutein in different food sources were excluded from this review. The collected publications were further examined to identify related studies and duplicates were removed. Out of the 253 initial collected studies, 119 were selected and included in the present review.

## 2. Dietary Sources of Zeaxanthin

As seen in Table 1, the overall occurrence of zeaxanthin in natural food products is low. The broad majority of xanthophyll-rich foods contain more lutein than zeaxanthin. Moreover, many of the zeaxanthin-containing products listed in Table 1 are not commercially available around the world. Consequently, the most predominant sources of zeaxanthin present in the human diet are corn-based foods along with pepper and egg yolk [33].

In what concerns the origin of the zeaxanthin-containing foods, plant-based foods are unequivocally the most investigated foods, as they are also more abundant in nature. In vegetables, zeaxanthin is present in its free form, while in ripped fruits it usually occurs in a more stable and less soluble form, i.e., esterified with various fatty acids [66,67]. After the ingestion of these zeaxanthin-rich fruits, the mono- or di-esters need to be enzymatically hydrolyzed into their free form in the gastrointestinal tract before absorption by the intestinal cells [68]. Some fruits with distinguished zeaxanthin content such as goji (*Lycium barbarum* L.) berries and sea buckthorn (*Hippophae rhamnoides* L.) berries have been studied in terms of zeaxanthin content and bioaccessibility [23,48,50,51] but a large number of exotic fruits with a high content of zeaxanthin still remain uninvestigated.

Animal-based food sources of zeaxanthin are limited and fully dependent on the animal’s diet. For instance, by supplementing the feed of laying hens, the content of both lutein and zeaxanthin in egg yolk can be enhanced [69,70,71]. Due to the high-lipid matrix, xanthophylls from egg yolk, present in a lipid-dissolved form, are more bioavailable than from plant-based sources [72].

Apart from plant and animal food sources, the dried edible biomass of microalgae constitutes a potential rich source of zeaxanthin. Several microalgae such as *Dunaliella* sp. and *Chlorella* sp. can accumulate impressive amounts of zeaxanthin (Table 1). Considering the steady increase in the human population and Earth’s limited resources, microalgae could be regarded as reliable sources of zeaxanthin and other beneficial byproducts in the near future. In addition to their less labor-intensive production and faster growing rate, the carotenoid content of microalgae is clearly superior to that of higher plants. Furthermore, microalgae can be amended through genetic engineering with the aim of improving the accumulation of high-value compounds such as carotenoids [73,74].

Food processing leads in most cases to a decrease in the content of zeaxanthin in varying degrees [27]. However, this slight disadvantage appears to be somewhat counterbalanced by a higher zeaxanthin bioaccessibility from the processed food than from its raw state [22]. In the case of microalgae, processing represents a critical step as it facilitates the disruption of the cellulose-rich wall of some microalgal strains, which further translates into an enhanced bioaccessibility of valuable bioactive compounds [62].

As previously mentioned, the intake of zeaxanthin is significantly lower as opposed to lutein and since frequently consumed fruits and vegetables such as apples, oranges, tomatoes and potatoes have a naturally low content of zeaxanthin, unexplored or novel foods are of utmost importance.

Even so, research solely on potential dietary sources of zeaxanthin is not sufficient. Information regarding zeaxanthin content must be coupled with the investigation of this xanthophylls digestive fate from different food matrices in order to promote adequate food sources for a zeaxanthin-enhanced absorption.

## 3. Zeaxanthin Bioaccessibility

Following the publication of the INFOGEST^®^ harmonized simulated digestion method [32], various research groups investigated carotenoid bioaccessibility from different food sources, some of them containing zeaxanthin. Table 2 summarizes the bioaccessibility of zeaxanthin from dietary sources obtained through the above-mentioned protocol. It should pointed out that even though the bioaccessibility was obtained using the same simulated digestion technique, each study was amended with consideration to the particularities of the tested food samples (as can be seen in the observations section), having carotenoid bioaccessibility as their common research purpose. Due to the matrix complexity not only of the foods of different origin (plant, animal and microalgal) but also amidst foods of the same origin (for instance, the plant-food sources), following an identical approach is unfeasible. Therefore, in the recent investigations presented in Table 2 the much-needed guidelines offered by the standardized in vitro procedure were applied after careful consideration of each selected food sample. In view of the fact that the in vivo process of carotenoid esters deacylation is not fully elucidated [75] and that the predominant form of zeaxanthin in human plasma is the free form [76], only the bioaccessibility of free zeaxanthin has been reviewed in the present paper.

Similar to its occurrence, the bioaccessibility of zeaxanthin from commonly consumed foods is relatively low (Table 2). Even though some of the analyzed food sources have a good zeaxanthin bioaccessibility, they are not frequently consumed not only because of the information gap between scientific evidence and consumers, but also because of their already emphasized unavailability in different regions of the world.

The release from the food matrix (also known as liberation) represents one of the many factors that affect carotenoid bioaccessibility, and consequently their bioavailability. Thermal processing promotes the release of zeaxanthin [77], as well as its solubilization into the aqueous environment of the stomach. The use of energy-saving high-pressure homogenization on raw mandarin juice exhibited an approximately ten-fold increase in zeaxanthin bioaccessibility as opposed to traditional pasteurization methods [78]. Similar results were observed in the case of orange juice, with a five-fold increase in zeaxanthin bioaccessibility [79].

Zeaxanthin-containing foods co-ingested with a source of fat stand a higher chance of solubilization and incorporation into mixed micelles [80]. It is for this reason that, for example, the bioaccessibility of zeaxanthin from sea buckthorn oil (*Hippophae rhamnoides* L.) [51] is significantly higher than that from *Pouteria lucuma* fruits [56] (Table 2). The food matrix in which zeaxanthin is delivered to the gastrointestinal tract is of paramount importance. Indeed, oil and other food products that contain a high amount of lipids have a superior zeaxanthin bioaccessibility compared to fruits in which the xanthophyll deposition restrains its release. Including dietary fat in the simulated digestion along with the investigated food sample has been shown to enhance the bioaccessibility of zeaxanthin among other carotenoids. By way of example, the addition of coconut oil (1%) in the in vitro digestion of goji berries boosted zeaxanthin bioaccessibility from 6.7% to 13.3% [23]. In the same perspective, fruits that have a natural high content of lipids such as the fruit of murici (*Byrsonima crassifolia*) have a higher zeaxanthin bioaccessibility [52].

Corn (*Zea mays* L.), food source from which the name zeaxanthin is derived, is considered one of the best dietary contributors of this xanthophyll. However, in recent a study focusing on the in vitro digestion of corn-based products, the bioaccessibility of lutein from boiled kernels and porridge was similar to that of zeaxanthin and even higher in the case of tortilla (22.4% versus 18.5%) [22]. This is an important aspect considering that the content of lutein in tortilla was 6.5-fold higher than zeaxanthin and more than 7-fold higher in boiled kernels and porridge, thus making corn a more powerful source of lutein than zeaxanthin.

The superior bioaccessibility of zeaxanthin from egg yolk is widely acknowledged [81]. Nevertheless, the contribution of egg yolk to the dietary intake of zeaxanthin is rather low. Considering that the zeaxanthin content in a boiled egg yolk with an average weight of 17 g is 11.8 μg/g with 90% bioaccessibility [81], the actual zeaxanthin absorption after the ingestion of a single boiled egg yolk would be 180.54 μg. In order to cover the 2 mg of zeaxanthin needed for a significant reduction in the progression of age-related macular degeneration [82], the ingestion of 11 egg yolks would be required. An alternative approach for the enhancement of dietary zeaxanthin would be to consume food sources containing a high content of zeaxanthin with a moderate to high bioaccessibility rather than highly bioaccessible food products with low zeaxanthin content.

Investigation on zeaxanthin bioaccessibility from processed beverages is also worthwhile seeing that most of them are commercially available and ready for consumption. In a broad study including twenty-two commercial milk-fruit beverages the bioaccessibility of zeaxanthin was found in the range of approximately 10% to 90%, with a mean percentage of 45.3% [83]. This wide range can be explained by the vastly different characteristics of each beverage comprising various types of fruits. A range of 7.4%–15.2% was observed for zeaxanthin bioaccessibility from different homemade cajá frozen pulp based beverages depending on the presence of other ingredients such as sugar and fat in the matrix [84]. In this case, the bioaccessibility of zeaxanthin increased in accordance with the presence and the amount of both sugar and fat.

Limited data is available on the bioaccessibility of zeaxanthin from microalgal sources. These microorganisms can produce strikingly high amounts of natural high-value byproducts such as zeaxanthin and the evaluation of their bioaccessibility after human ingestion represents an interesting yet uninvestigated area of research. In addition to cell disruption, systems such as oil-in-water emulsions prepared from the extracted microalgal oil can provide an increased zeaxanthin bioaccessibility [61].

Aside from its importance in the assessment of bioavailability, bioaccessibility represents a key factor in designing food formulations that aim at maximizing the absorption of a particular bioactive compound. Recently, yogurt was used as a delivery system for zeaxanthin nanoparticles and nanoemulsions prepared from a natural goji berries extract [85] and the bioaccessibility of zeaxanthin was 3.66% and 4.46%, respectively. As the consumer preferences have shifted towards more natural and organic food products, the addition of zeaxanthin extracted from rich food sources instead of synthetic zeaxanthin to potentially efficient dietary carriers represents a promising alternative and a great opportunity for the food industry.

The design of carotenoid formulations compatible with food or pharmaceutical applications represents an important strategy to improve the stability and bioaccessibility of these lipophilic pigments [68,86,87]. The most common colloidal delivery systems for carotenoids are liposomes, emulsions (micro- and nano-), solid lipid nanoparticles and microgels [86,87]. Nanoencapsulation of carotenoids within lipid-based carriers ensures the safe passage of carotenoids throughout the gastrointestinal tract and their subsequent release at the intended sites [86].

Although further in vivo studies are necessary for validation, the results obtained using the standardized in vitro digestion method constitute an important preliminary screening of zeaxanthin bioaccessibility from different food matrices. The scope of this information gathered through collective efforts of the scientific community is to gain insight on the barriers that restrict intestinal absorption in an easily reproducible, less expensive and less labor-intensive manner than human nutritional studies. Table 3 comprises some relevant studies on zeaxanthin bioaccessibility that have employed other in vitro digestion methods and therefore a direct comparison is not recommended. Nevertheless this previously achieved data should not be disregarded as it constitutes an important direction for the path forward in finding more bioaccessible zeaxanthin sources.

Further research looking into the bioaccessibility of zeaxanthin from unexamined food sources is required with the purpose of developing a database available to the population vulnerable or already affected by age-related ophthalmic disorders.

## 4. Zeaxanthin and Health Related Benefits

Due to its accumulation in the human retina, zeaxanthin is known primarily as one of the three macular pigments. Zeaxanthin and *meso*-zeaxanthin are predominantly distributed near the fovea, whereas lutein is more concentrated in the peripheral retina [12,97]. In contrast to other mammals, the primate’s carotenoid cleavage enzyme BCO2 has a structural particularity that reduces its ability to capture and to cleave xanthophylls. Consequently, only primates can accumulate lutein and zeaxanthin in their retina [97]. In recent decades, lutein and zeaxanthin have been associated with a reduced risk of developing several ocular diseases such as age-related macular degeneration and cataract [98,99]. Owning to the high levels of oxygen along with high concentrations of polyunsaturated fatty acids and exposure to visible light, human retina is prone to oxidative damage. The photoprotective effect of the macular pigments is related to their ability to act as filter pigments through the absorption of high-energy light (blue light) [100]. Moreover, their capacity to neutralize reactive oxygen species (e.g., singlet oxygen, hydroxyl radical, superoxide anion) is essential for limiting the oxidative stress-induced damage such as lipid peroxidation, protein oxidation, DNA damage and cytotoxic lipofuscin accumulation [97,101]. In cultured human retinal pigment epithelial cells, zeaxanthin and lutein enhanced both the concentration of reduced glutathione and the activity of superoxide dismutase and glutathione peroxidase [102].

As zeaxanthin cannot be synthesized by humans, its serum concentration is strictly related to the diet and its intake varies depending on age, sex and ethnicity. Generally, the intake of zeaxanthin is lower than that of lutein and in a study conducted on the US population, the relative zeaxanthin to lutein ratio decreased with age, being lower in females and higher in Mexican Americans. Additionally, lower zeaxanthin to lutein ratios were found in groups with a higher risk of developing age-related macular degeneration (i.e., older participants) [103]. Both low macular pigment optical density (MPOD) and low serum xanthophylls have been previously associated with an increased susceptibility to age-related macular degeneration (AMD) [104,105]. Although contradictory results have been reported in various studies concerning the efficiency of dietary supplementation with macular pigments for the prevention of AMD, valuable findings were provided by the AREDS2 study. The addition of lutein (10 mg), zeaxanthin (2 mg) and omega-3 fatty acids (1 g) to the original AREDS formula determined a reduction of 26% in risk of the progression of advanced AMD compared to the aforementioned formula (for the quintile with the lowest intake) [106]. Furthermore, lutein/zeaxanthin supplementation could also be associated with a lower risk of progression to cataract surgery [107].

Based on their preferential accumulation in the human brain and the acknowledged correlation between MPOD and brain carotenoids, lutein and combinations of lutein and zeaxanthin have been investigated for their contribution in cognitive function. Zeaxanthin concentration in the brain tissue of centenarians decedents was significantly correlated with premortem memory retention, verbal fluency and dementia [108]. In addition to a significant increase in MPOD, several cognitive parameters such as complex attention and cognitive flexibility were improved in both older women and men (mean age 72.51 years) after twelve months supplementation with 10 mg of lutein and 2 mg of zeaxanthin, with the composite memory being improved only in men [109].

Lutein and zeaxanthin were among the major carotenoids found in the infant brain and the detection of higher concentrations of lutein and zeaxanthin in almost all the brains of term infants as opposed to preterm infants may indicate an important role in cognition [110].

Similarly, better cognitive performance and neural efficiency were observed in children with higher MPOD [111]. Lutein and zeaxanthin seem to play an important role in pre-natal and post-natal development, as suggested by their presence in cord blood and incipient macula in human fetuses [8]. Henriksen et al. [112] found a correlation between zeaxanthin concentration in serum and MPOD in healthy term infants, as well as a correlation between the mother’s zeaxanthin concentration in serum and infant MPOD. These results indicated that maternal zeaxanthin has a more relevant role in macular pigment deposition in utero than lutein. As breast milk is the only source of lutein and zeaxanthin for young infants, the prospect of maternal supplementation and the development of macular pigment-fortified milk formulas are of great interest [8].

The blue light-filtering ability and the antioxidant properties of zeaxanthin have also been proven to have an important role in skin protection. Several studies on cell cultures and animal models displayed the protective effect of lutein and zeaxanthin through cell viability improvement, inhibition of matrix metalloproteinases (MMPs) and of inflammation and immunosuppression associated with UV-induced oxidative damage [113]. After twelve weeks of supplementation with 5 mg of lutein and 0.3 mg of zeaxanthin (in capsule form, twice per day), a significant reduction in lipid peroxidation associated with an improvement in skin hydration, skin lipid content, elasticity and photoprotection has been observed in women exhibiting signs of premature aging [114].

Recently, Christensen et al. [115] used the data reported by the 2003–2014 National Health and Nutrition Examination Survey (NHANES) to investigate cross-sectional associations between dietary and serum levels of carotenoids in relation to non-alcoholic fatty liver disease (NAFLD). The carotenoid intake was estimated by a 24-h recall and for some groups the serum carotenoids were measured by HPLC. A lower intake of carotenoids (including zeaxanthin) was observed for NAFLD subjects. Moreover, a higher level of serum carotenoids has been associated with a reduced risk of developing NAFLD. Although rodents do not accumulate xanthophylls due to the high activity of BCO2 [9], a protective effect of zeaxanthin (free or esterified) against ethanol induced hepatic damage in animal models (rats, mice) and in nonalcoholic steatohepatitis (gerbils) was indicated in several studies reviewed by Murillo et al. [116].

The antioxidant activity of zeaxanthin has a major importance in limiting the oxidation of both HDL and LDL, thus contributing to the prevention of atherosclerosis and other associated cardiovascular diseases. A study carried out over the course of 18 months on 573 middle-aged healthy subjects at baseline revealed that the change in carotid intima-media thickness (IMT) was significantly inversely correlated with the serum concentration of lutein, zeaxanthin, β-cryptoxanthin and α-carotene [117]. Although zeaxanthin and β-carotene were negatively correlated with right common carotid artery stiffness, elastic modulus and pulse wave velocity in subjects with early atherosclerosis, no statistical differences were observed as regards zeaxanthin serum concentration of the cases and the controls [118].

Considering that eggs constitute rich sources of highly bioavailable lutein and zeaxanthin, a supplementation study with 1 soft boiled egg per day for 4 weeks was conducted in moderately hypercholesterolemic Japanese males in order to investigate its effect on LDL oxidation. Despite the higher cholesterol intake, the total cholesterol level was not affected by the egg supplementation and an increase in both lutein and zeaxanthin serum concentration was observed. In addition, a decrease in malondialdehyde modified low-density lipoprotein concentrations and a prolonged LDL oxidation lag were recorded, emphasizing the antioxidant protection of these xanthophylls [119].

## 5. Conclusions

As age-related macular degeneration (AMD) is one of the leading causes of blindness, seeking bioaccessible natural sources of macular xanthophylls represents the way forward in preventing and delaying the progression of this medical condition. The presence of lutein and zeaxanthin in the infant brain further indicates an important role of these dihydroxycarotenoids in cognitive function, also confirmed by the lower concentrations found in elderly with mild cognitive impairment.

Along with some zeaxanthin-rich exotic fruits, the edible biomass of microalgae emerges as a promising zeaxanthin source and deserves further investigation.

This brief overview of potentially bioaccessible food sources of zeaxanthin provides a valuable support not only for the industry in the development of functional foods designed so as to enhance the intake of this oxygenated carotenoid, but also for nutritionists and end-consumers in the wise selection of dietary sources with an elevated zeaxanthin absorption.

## Figures and Tables

**Table 1:** Dietary sources of zeaxanthin (μg/g dry weight ^a^ or μg/g fresh weight ^b^).

Plant Sources	Zeaxanthin	Ref.
Einkorn wheat (*Triticum monococcum*)	0.94 ^a^	[34]
Khorasan wheat (*Triticum turgidum* subsp. *turanicum*)	0.71 ^a^	[34]
Durum wheat (*Triticum turgidum* subsp. *durum*)	0.49 ^a^	[34]
Corn (*Zea mays* L.)	10.31 ^a^	[34]
Corn flakes	1.02–2.97 ^a^	[35]
Corn chips	1.05 ^a^	[36]
Corn tortilla	0.93 ^a^	[36]
Corn masa	1.13 ^a^	[36]
Corn flour	9.4 ^a^	[37]
Boiled corn	3.7 ^a^	[37]
Potato (*Solanum tuberosum* L.)	7.7 ^a^	[37]
Sweet potato (*Ipomoea batatas*)	0.3 ^a^	[37]
Squash (*Cucurbita maxima*)	1.9 ^a^	[37]
Kidney been (*Phaseolus vulgaris* L.)	0.1 ^a^	[37]
Okra (*Abelmoschus esculentus*)	0.1 ^a^	[37]
Beet (*Beta vulgaris* L.)	0.7 ^a^	[37]
Tomato (*Solanum lycopersicum* L.)	1.3 ^a^	[37]
Hot chili peppers (*Capsicum frutescens* L.)	1230 ^a^*	[38]
Pepper (*Capsicum annuum* L.)		
red	55.0–97.0 ^a^	[39]
green	1.7–5.7 ^a^	[39]
orange	62.0 ^a^	[37]
yellow	4.4 ^a^	[37]
India mustard (*Brassica juncea*)	0.8 ^a^	[37]
Watercress (*Nasturtoum officinale*)	0.4 ^a^	[37]
Endive (*Cichorium endivia* L.)	0.5 ^a^	[37]
Romaine lettuce (*Lactuca sativa* L. var. *longifolia*)	0.7 ^a^	[37]
Lettuce (*Lactuca sativa* L.)	0.1 ^a^	[37]
Cabbage (*Brassica oleracea* L.)	0.1 ^a^	[37]
Spinach (*Spinacia oleracea* L.)	0.7 ^a^	[37]
Kale (*Brassica oleracea* L. var. *sabellica*)	163–2460 ^a^	[40]
Zucchini blossoms (*Cucurbita pepo* L.)	32.7 ^b^*	[41]
Artichoke heart (*Cynara cardunculus* L. var. *scolymus*)	0.18 ^b^	[33]
Avocado (*Persea americana*)	0.08–0.18 ^b^	[42]
Apple (*Malus domestica*)		
flesh	nd– 0.04 ^a^	[43]
peel	nd–0.52 ^a^	[43]
Apricot (*Prunus armeniaca* L.)	nd–0.39 ^b^	[44]
European plum (*Prunus domestica* L.)	0.1 ^a^	[37]
Nectarine (*Prunus persica*)	0.2 ^a^	[37]
Orange ^‡^ (*Citrus sinensis*)	0.3 ^a^	[37]
Orange juice ^‡^ (*Citrus sinensis*)	0.1 ^a^	[37]
Grafted orange ^‡^ (*Citrus sinensis*)	1.1 ^a^	[37]
Grafted orange (juice) ^‡^	0.6 ^a^	[37]
Mandarin ^‡^ (*Citrus reticulata*)	2.1 ^a^	[37]
Mandarin juice ^‡^ (*Citrus reticulata*)	1.7 ^a^	[37]
Red grapefruit ^‡^ (*Citrus paradisi*)	0.2 ^a^	[37]
Peruvian groundcherry (*Physalis peruviana* L.)	0.4 ^a^	[37]
Strawberry tree (*Arbutus unedo* L.) fruits	0.7–2.0 ^a^	[45]
Raspberry (*Rubus idaeus* L.)	0.14–0.49 ^a^	[46]
Rose hip (*Rosa* spp.)	23–107 ^a^*	[47]
Wolfberry (goji berry) (*Lycium barbarum* L.)	1231.1 ^a^*	[48]
Red Chinese lantern fruit (*Physalis alkekengi* L.)	847–1035 ^a^*	[49]
Sea buckthorn (*Hippophae rhamnoides* L.)		
berries	193–424 ^a^*	[50]
oil (cold-pressed)	2312.2 ^b^*	[51]
Murici fruit (*Byrsonima crassifolia*)	5.4 ^a^*	[52]
Arazá fruit (*Eugenia stipitata*)		
peel	1.14 ^b^	[53]
pulp	0.17 ^b^	[53]
Astringent persimmon (*Diospyros kaki* Thunb. var. Rojo brillante)	10.2 ^b^*	[54]
Cashew apples (*Anacardium occidentale* L.)		
peel	0.51–2.69 ^b^*	[55]
pulp	0.04–0.58 ^b^*	[55]
Corozo ^‡^ (*Aiphanes aculeata*)	79.2 ^a^	[37]
South American sapote ^‡^ (*Quararibea cordata*)	46.2 ^a^	[37]
Passion fruit ^‡^ (*Passiflora edulis*)	0.2 ^a^	[37]
Mango ^‡^ (*Mangifera indica*)	0.5 ^a^	[37]
Red papaya ^‡^ (*Carica papaya*)	0.6 ^a^	[37]
Yellow guava ^‡^ (*Psidium guajava* L.)	0.2 ^a^	[37]
Pineapple ^‡^ (*Ananas comosus*)	0.1 ^a^	[37]
Melon ^‡^ (*Cucumis melo* L.)	0.1 ^a^	[37]
Tahitian apple ^‡^ (*Spondias dulcis*)	0.1 ^a^	[37]
Cassabanana ^‡^ (*Sicana odorífera*)	0.4 ^a^	[37]
Tree tomato ^‡^ (*Cyphomandra betacea*)	1.7 ^a^	[37]
Red tree tomato ^‡^ (*Cyphomandra betacea*)	2.4 ^a^	[37]
Roselle ^‡^ (*Hibiscus sabdariffa* L.)	0.8 ^a^	[37]
Membrillo ^#^ (*Gustavia superba*)	37.6 ^a^	[37]
Canistel ^#^ (*Pouteria campechiana*)	19.7 ^a^	[37]
Chinese passion fruit ^#^ (*Cionosicyos macranthus*)	2.8 ^a^	[37]
Sastra ^#^ (*Garcinia intermedia*)	84.7 ^a^	[37]
Yellow mombin ^#^ (*Spondias mombin* L.)	1.2 ^a^	[37]
Guanabana toreta ^#^ (*Annona purpurea*)	6.8 ^a^	[37]
Purple mombin ^#^ (*Spondias purpurea* L.)	0.8 ^a^	[37]
Chinese rose ^#^ (*Pereskia bleo*)	0.8 ^a^	[37]
Nance ^#^ (*Byrsonima crassiflora*)	0.2 ^a^	[37]
Lucuma fruit (*Pouteria lucuma*)		
Molina variety	3.44–5.76 ^b^*	[56]
Beltran variety	5.74 –6.66 ^b^*	[56]
Sarsaparilla (*Smilax aspera* L.) berries	8.56 ^b^*	[57]
Animal sources		
Butter	nd - 0.02 ^b^	[58]
Marine crab (*Charybdis cruciata*)		
meat	0.02 ^b^	[59]
Freshwater crab (*Potamon potamon*)		
meat	1.72 ^b^	[59]
Eggs		
raw	1.5 ^a^	[60]
boiled	1.3 ^a^	[60]
poached	1.3 ^a^	[60]
omelette	1.14 ^a^	[60]
Microalgal sources		
*Nannochloropsis* sp.		
suspension	420 ^a^	[61]
oil	1930 ^b^	[61]
*Chlorella ellipsoidea*	1999 ^a^	[62]
*Dunaliella salina*	11270 ^a^	[63]
*Phaeodactylum tricornutum*	679.2 ^a^	[64]
*Scenedesmus almeriensis*	370 ^a^	[65]

nd, not detected; *, zeaxanthin + zeaxanthin mono- and diesters; ^#^, Panamanian wild fruit; ^‡^, fruit cultivated in Panama.

**Table 2:** Recent research (last 5 years) with regard to zeaxanthin bioaccessibility (%) from different food sources assessed through the internationally recognized in vitro digestion method [32].

	Food Matrix	Bioaccessibility (%)	Ref.	Observations
	Sea buckthorn (*Hippophae rhamnoides* L.)		[51]	The oral phase was not considered and porcine cholesterol esterase was included in the protocol.
	oil	61.5		
	oil-in-water (o/w) emulsion	64.6		
Plant sources	Goji berries (*Lycium barbarum* L.)	13.3	[23]	The tested food sample (dried goji berries) was supplemented with 1% (*w/w*) coconut fat.
	Astringent persimmon (*Diospyros kaki* Thunb, var. Rojo Brillante)	2.5	[54]	The persimmon samples were subjected to a high hydrostatic pressure treatment and the protocol was slightly amended as concerns the simulated digestion fluids.
	Cajá (*Spondias mombin* L.) water and milk based beverages	7.4–15.2	[84]	Six homemade cajá frozen pulp based beverages were analyzed through the slightly adjusted protocol.
	Ortanique mandarin juices (*Citrus reticulata* x *Citrus sinensis*)	8.8–82	[78]	Five mandarin juices subjected to traditional pasteurization and energy-saving high-pressure homogenization treatments were analyzed through the slightly adjusted protocol in which the oral phase was not considered.
	Orange juice (*Citrus sinensis* L. Osb.)	16–79	[79]	Five orange juices subjected to traditional pasteurization, energy-saving high-pressure homogenization and a combined centrifugation and homogenization technique were analyzed through the slightly adjusted protocol in which the oral phase was not considered.
	Commercial milk-fruit juice beverages	45.3	[83]	Twenty-two commercial milk-fruit juice beverages were analyzed through the slightly adjusted protocol. The oral phase was not considered and the bioaccessibility of zeaxanthin was expressed as mean percentage of the twenty-two commercial beverages investigated.
	*Pouteria lucuma* fruits		[56]	Two varieties of seedless lucuma fruit pulps were analyzed through the slightly adjusted protocol.
	variety “Molina”	5.8		
	variety “Beltran”	1.6		
	Murici (*Byrsonima crassifolia*) fruit	22	[52]	The freeze-dried murici fruit were rehydrated and analyzed through the slightly adjusted protocol along with other reported in vitro digestion methods.
	Maize (*Zea mays* L.)		[22]	After their preparation from maize, boiled kernels, porridge and tortilla were analyzed through the slightly adjusted protocol. In the case of porridge, the oral phase was not included.
	boiled kernels	2.4		
	porridge	7.8		
	tortilla	18.4		
Animal sources	Egg yolk (hard boiled)	90	[81]	The yolk of hard-boiled commercial eggs was analyzed through the slightly adjusted protocol along with another in vitro digestion method.
	Egg yolk		[60]	The protocol was amended so as to simulate the digestion conditions of exocrine pancreatic insufficiency patients.
	boiled	26–98		
	poached	28–103		
	omelette	31–111		
Microalgal sources	*Nannochloropsis* sp.		[61]	*Nannochloropsis* sp. (untreated biomass, high pressure homogenized biomass and oil-in-water emulsion) was analyzed through the slightly adjusted protocol. The oral phase was not considered and the results are expressed in terms of micellar incorporation (%).
	Untreated suspension	9		
	HPH suspension	19		
	o/w emulsion	54		

**Table 3:** Relevant research on zeaxanthin bioaccessibility (%) from different food sources assessed through various in vitro digestion methods.

	Food Matrix	Bioaccessibility (%)	Ref.
Plant sources	Boiled yellow-fleshed potato (*Solanum tuberosum* L.)	55–71	[88]
	Clementine mandarins (*Citrus* x *clementina*)		[89]
	pulp	14.1–27.2	
	juice	65.9	
	Spinach (*Spinacia oleracea* L.)	6.7	[90]
	Lettuce (*Lactuca sativa* L.)	5.7	
	Sweet corn (*Zea mays* L.)	54	
	Red pepper (*Capsicum annuum* L.)	48.4	
	Orange (*Citrus sinensis*)	38.9	
	Jalapeño peppers (*Capsicum annuum* L.)		[91]
	brown peppers	87.1	
	50% red peppers	59.3	
	75% red peppers	47.4	
	Pungent Peppers (*Capsicum annuum* L.)		[92]
	green pepper	75.6	
	red pepper	72.9	
	Red chili peppers (*Capsicum annuum* L.)		[93]
	fresh	0–74.5	
	frozen	23.9–90	
	boiled	0–93.4	
	Processed milk- and soy-based fruit beverages		[94]
	whole milk-fruit beverages	30.2–71.2	
	skimmed milk-fruit beverages	7.5–35.2	
	soy milk-fruit beverages	29.9–100.5	
	Honeydew melon (*Cucumis melo* L.)	50.2	[95]
Animal sources	Egg yolk	91	[81]
Microalgal sources	*Arthrospira* sp.	4.9	[96]
	*Phaeodactylum tricornutum*	29	[64]
	*Chlorella ellipsoidea*		
	Untreated	2.6	[62]
	Microfluidized at 5000 psi	7.8	
	Microfluidized at 10,000 psi	22	
	Microfluidized at 20,000 psi	32.6	
